# Carnivorous and Herbivorous Types in Man

**Published:** 1915-05

**Authors:** 


**Carnivorous and Herbivorous Types in Man,** John Bryant, Boston, "Boston Medical and Surgical Journal," March 4, 1915. Body form influences intestinal length, regardless of food habits (Is not this statement reversed?) Intestinal length varies with species, regardless of food habits. But, in a given species, there may be a variation of 100% in individuals. This variation depends mainly on digestibility of food. Offspring of the same female animal have approximately equal intestinal length at birth (Is not this generally true of the young of a given species?) In an adult, body form and intestinal length depend mainly on heredity, not sex. Carnivorous animals may become facultatively herbivorous and vice versa. In carnivorae, the body form is long and thin, the intestine short and the small intestine longer than the large. In herbivorae the first two statements are reversed and the small intestine approaches or may equal the large in length. The human intestine approaches the carnivorous type but variations take place to the extent of 100%.

Biliary contrasts: Herbivorous (this word and carnivorous represented by initials subsequently) large amount of bile; C. small. Cholesterin passes through liver to bile in H. while in C. it filters through liver into blood, only a small amount being excreted in bile. Cirrhosis of liver in H. is usually marked by enlargement; in C. by contraction. The gall bladder of H. is small, muscular and thick; in C. strophic, large, thin, ptotic. Glycocholic acid is scanty in H., abundant in C. Taurocholic acid same. Gall stones are oftener of pure cholistering in H., always due to infection (chronic or mitigated) in C.

Cardiorenal phenomena. Blood pressure higher, tendency to apoplexy greater through arterio-sclerosis, pulse faster and fuller in H. The heart is larger and more transverse, the diaphragm higher, aorta larger in H. Red corpuscles are more numerous in H., lymphocytes, eosinophiles and tendency to leucocythaemia greater in C. Status lymphaticus limited to C.

Chemic phenomena (stated to be known for lower animals and probably true for man). Acidosis more fatal to H. which do not form ammonia from proteids as do C. Calcium in urine greater in H. Cholesterin greater in blood in H. Faeces more copious, lighter in color, acid from fermentation in H., tendency to alkaline putrefaction in C. Glycocholic acid greater in bile in C.; opposite for taurocholic acid. Hippuric acid in urine regularly in H., usually absent in C. Indoxyl, phosphates, urea and uric acid much higher in C. Urinary reaction normally alkaline or neutral in H., acid on C. Urinary acidity not limited in H., limited in C. (This statement needs qualification.) This tabular review is followed out to great length and we refer to the original article for the remainder.

